# Acute Pyelonephritis with Bacteremia Caused by *Enterococcus hirae*: A Rare Infection in Humans

**DOI:** 10.1155/2016/4698462

**Published:** 2016-04-05

**Authors:** Ana Pãosinho, Telma Azevedo, João V. Alves, Isabel A. Costa, Gustavo Carvalho, Susana R. Peres, Teresa Baptista, Fernando Borges, Kamal Mansinho

**Affiliations:** ^1^Egas Moniz Hospital, Department of Internal Medicine, 1349-019 Lisbon, Portugal; ^2^Egas Moniz Hospital, Infectious and Tropical Diseases Department, 1349-019 Lisbon, Portugal; ^3^Cascais Hospital, Internal Medicine Department, 2755-009 Alcabideche, Portugal

## Abstract

Enterococci are one of the usual residents of the microflora in humans. In the last decade this genus has been reported as the third most common cause of bacteremia. We present the case of a 78-year-old female who was admitted to the emergency room because of nausea, lipothymia, and weakness. She was diagnosed with a pyelonephritis with bacteremia, with the isolation in blood and urine cultures of* Escherichia coli* and* Enterococcus hirae*. This last microorganism is a rarely isolated pathogen in humans. Currently it is estimated to represent 1–3% of all enterococcal species isolated in clinical practice.

## 1. Introduction

Enterococci were initially part of the* Streptococcus* genus. It was not until 1984 that the* Enterococcus* genus was first described by Schleifer and Kilpper-Balz. Many of the members of this genus make up the resident microflora of humans [1].* Enterococcus faecalis* (80%) and* Enterococcus faecium* (10%) are frequently associated with human infection such as bacteremia, endocarditis, and urinary tract infections. In the last decade Enterococci have been reported as the third most common cause of bacteremia [2, 3].

*Enterococcus hirae* accounts for less than 1% of enterococcal species isolated in human clinical samples. We describe a case of acute pyelonephritis with bacteremia in a 78-year-old woman.

## 2. Case Presentation

A 78-year-old female with a personal history of atrial fibrillation and chronic renal disease was admitted to the emergency room because of nausea, lipothymia, and generalized weakness. On examination the patient was oriented, vitally stable, and apyretic. There were no significant findings in the neurologic examination and the rest of the physical exam was unremarkable.

Initial laboratory findings showed an elevation of inflammatory markers with a white blood cell count of 16,400/*μ*L with left shift (neutrophil 85,9%), C-reactive protein of 28 mg/dL, hemoglobin of 13 g/dL, platelet count of 147,000/microL, serum creatinine of 1,15 mg/dL, and urea of 75 mg/dL. Urinalysis showed leucocituria with negative nitrites and many of leucocytes. Chest X-ray, electrocardiography, and renal echography were unremarkable.

Having admitted an uncomplicated pyelonephritis, the patient was put on empirical antibiotherapy with amoxicillin-clavulanic acid after urine and blood cultures were obtained.

On the third day of antibiotherapy the patient remained afebrile and showed improvement of the laboratory findings and symptoms. The urine cultures identified* Escherichia coli* resistant to trimethoprim-sulfamethoxazole, cefalotin, and amoxicillin-clavulanic acid but sensitive to piperacillin-tazobactam. They also showed* Enterococcus hirae* resistant to cefuroxime and nitrofurantoin but susceptible to amoxicillin-clavulanic acid and piperacillin-tazobactam. This last bacterium was also isolated in the blood culture, presenting the same sensitivity profile. Due to the resistance patterns of both microorganisms we decided to change the antibiotic to piperacillin-tazobactam. Treatment options were discussed with the Microbiology Department: given the fact that there was the isolation of a multiresistant* E. coli* strain and the patient was clinically improving, the antibiotherapy was maintained, and a total of 14 days of piperacillin-tazobactam was completed.

Upon identification of the* Enterococcus hirae* a more detailed epidemiological interview was conducted. The patient mentioned having had contact with farm animals such as birds, namely, parrots, dogs, horses, and cats a month before, while staying in a country house.

## 3. Discussion

*Enterococcus hirae* is a pathogen frequently associated with infections in animal species, particularly in psittacine birds, cats, and rats [4, 5]. The first report of human infection by this agent was described by Gilad et al. in 1998 [6] in a patient with end-stage renal disease, undergoing hemodialysis, and presenting with septicemia [7].

According to most reviews, the prevalence of nonfaecalis and nonfaecium Enterococci ranges from 2 to 10% [8].

To the best of our knowledge, there are only eleven reports describing human infection in the literature [9] (Table 1). Amongst the cases described are infections of native and prosthetic valves, acute pyelonephritis, septicaemia, and spondylodiscitis.

Our case is the fourth case of acute pyelonephritis with bacteremia and the twelfth, worldwide, reported case of established human infection caused by* Enterococcus hirae*.

Enterococci are relatively resistant to many antibiotics that are active against Gram-positive cocci, including cephalosporins, macrolides, and clindamycin. Penicillins and glycopeptides have the best* in vivo* activity. However, ampicillin typically has greater* in vitro* killing ability than vancomycin. Enterococci have an intrinsic low-level resistance to the aminoglycosides due to the decreased ability of these agents to penetrate the cell wall. This can be overcome by the addition of cell wall-active agents (such as penicillins and glycopeptides) resulting in a synergistic killing effect [8].

The true incidence of the infections caused by this agent may be underestimated because of the misidentification of some species due to the exhibition of aberrant sugar reactions by some Enterococci or due to lack of application of the appropriate tests to identify rare species of Enterococci [8]. This finding is of some concern. A study conducted in a tertiary South Indian hospital investigated the prevalence of unusual and atypical species of Enterococci causing human infections. Forty-three percent of the isolates were from cases of septicemia, which illustrates the virulence of these species [8]. It is, thus, important to raise awareness of these rare pathogens in order to increase their detection and prompt the introduction of accurate antibiotherapy guided, whenever possible, by the susceptibility profile.

*E. hirae* is a rarely isolated pathogen in humans but it is underreported due to misidentification. Currently it is estimated to represent 1–3% of all enterococcal species isolated in clinical practice [12]. In our case, there was a clear epidemiological context in which our patient had contact with birds, the species most often affected by this pathogen.

## Figures and Tables

**Table 1:** Reported cases of human infections due to *E. hirae*. Adapted from Alfouzan et al. [9]. AMC, amoxicillin-clavulanic acid; AMP, ampicillin; AMX, amoxicillin; CFZ, cefazolin; CHF, congestive heart failure; CIP, ciprofloxacin; CMZ, cefmetazole; CRO, ceftriaxone; DM, diabetes mellitus type 2; GEN, gentamicin; LVX, levofloxacin; LZD, linezolid; PTZ, piperacillin-tazobactam; RF, rifampicin; VAN, vancomycin.

Reference	Year	Age/sex	Diagnosis	Risk factor	Clinical sample	Treatment
Gilad et al. [6]	1998	48/M	Septicaemia	End-stage renal disease, hemodialysis	Blood	VAN
Park et al. [10]	2000	21/F	Acute pyelonephritis	None	Blood, urine	AMP
Poyart et al. [11]	2002	72/M	Native valve endocarditis	Coronary artery disease	Blood	AMP, GEN, RIF, VAN
Canalejo et al. [12]	2008	55/M	Spondylodiscitis	DM	Blood	Discectomy, AMP, GEN, LVX
Kim et al. [13]	2009	57/F	Acute pyelonephritis	Rheumatoid arthritis	Blood, urine	CIP, CRO, AMC
Talarmin et al. [14]	2011	78/F	Infective endocarditis	Bioprosthetic valve	Blood	AMX, GEN
Chan et al. [15]	2012	62/F	Acute pyelonephritis	None	Blood, urine	CFZ, GEN, AMP
Chan et al. [15]	2012	83/F	Acute cholangitis	CHF, valvular heart disease	Blood	CMZ
Sim et al. [16]	2012	61/M	Bacterial peritonitis	Cirrhosis, DM	Blood, ascitic fluid	AMP
Anghinah et al. [7]	2013	56/F	Infective endocarditis	DM, cardiac ablation due to arrhythmia, foramen ovale	Blood	AMP, RIF
Alfouzan et al. [9]	2014	48/M	Multiple splenic abscesses	DM	Blood, pus	Splenectomy, AMP, PTZ, LAZ
